# Tuning the Topography of Dynamic 3D Scaffolds through Functional Protein Wrinkled Coatings

**DOI:** 10.3390/polym16050609

**Published:** 2024-02-23

**Authors:** Elizabeth Oguntade, Daniel Fougnier, Sadie Meyer, Kerrin O’Grady, Autumn Kudlack, James H. Henderson

**Affiliations:** 1 Department of Biomedical & Chemical Engineering, Syracuse University, Syracuse, NY 13244, USA; eoguntad@syr.edu (E.O.); dfougnie@syr.edu (D.F.); ssmeyer@syr.edu (S.M.); kaogrady@syr.edu (K.O.);; 2 BioInspired Syracuse: Institute for Material and Living Systems, Syracuse University, Syracuse, NY 13244, USA

**Keywords:** thin film wrinkling, silk fibroin (SF) biopolymer, shape–memory polymers (SMPs), additive manufacturing, 4D printing, 3D cellular microenvironments

## Abstract

Surface wrinkling provides an approach to fabricate micron and sub-micron-level biomaterial topographies that can mimic features of the dynamic, in vivo cell environment and guide cell adhesion, alignment, and differentiation. Most wrinkling research to date has used planar, two-dimensional (2D) substrates, and wrinkling work on three-dimensional (3D) structures has been limited. To enable wrinkle formation on architecturally complex, biomimetic 3D structures, here, we report a simple, low-cost experimental wrinkling approach that combines natural silk fibroin films with a recently developed advanced manufacturing technique for programming strain in complex 3D shape–memory polymer (SMP) scaffolds. By systematically investigating the influence of SMP programmed strain magnitude, silk film thickness, and aqueous media on wrinkle morphology and stability, we reveal how to generate and tune silk wrinkles on the micron and sub-micron scale. We find that increasing SMP programmed strain magnitude increases wavelength and decreases amplitudes of silk wrinkled topographies, while increasing silk film thickness increases wavelength and amplitude. Silk wrinkles persist after 24 h in cell culture medium. Wrinkled topographies demonstrate high cell viability and attachment. These findings suggest the potential for fabricating biomimetic cellular microenvironments that can advance understanding and control of cell–material interactions in engineering tissue constructs.

## 1. Introduction

In vivo, the extracellular matrix (ECM) presents cells with micro- and nanoscale topographies that can modulate cell behavior in development, health, and disease [1,2,3,4,5]. In light of the importance of topography to cell function, micron and sub-micron scale topographies have been studied for their potential to modulate cellular behavior in tissue engineering strategies. A variety of topographical patterns, such as grooves [6], pillars [7], holes [6], and wrinkles [8,9,10,11,12,13], have been found to affect cellular responses including morphology, migration, alignment, and differentiation.

Wrinkles are topographical patterns found frequently in nature [14,15] and have been studied for their potential to mimic features of the ECM. A common approach to fabricating wrinkles is controlled compression of a stiff, thin film on a softer, compliant substrate in a bilayer material platform [14,15]. Using this approach in polymeric and inorganic material systems, wrinkles have been demonstrated to regulate diverse cell behaviors [8,9,10,11,12,13]. For example, Wang et al., developed a graphene oxide wrinkled surface on which human and murine fibroblasts had the capacity not only to adhere to the textured films but also adopted a morphology of pronounced alignment and elongation compared to cells on non-wrinkled, flat graphene controls [12]. In their work using a tunable gold wrinkle cell culture platform that could align embryonic stem cell-derived endothelial cells, Hatano et al. demonstrated the potential for rapid endothelialization of stents and vascular grafts [13].

Despite progress in understanding the effects of wrinkle topographies on cellular behavior, most thin film wrinkling research to date has used planar or two-dimensional (2D) substrates [8,9,10,11,12,13,14,15]. As a result, the buckling phenomenon that produces wrinkle topographies on planar or 2D surfaces is relatively well-studied and understood. In contrast, understanding of wrinkle formation on three-dimensional (3D) surfaces or arbitrarily complex 3D parts is limited. Li et al. created wrinkled surface patterns on various 3D microstructures via partial polymerization during photolithography to acquire a partially cured polymer layer on fabricated 3D microwell or micro-post arrays [16]. During plasma treatment, manipulation of the ultraviolet (UV) light exposure time enabled the resultant 3D wrinkled surface to be morphologically tuned and spatially regulated. Bovine ligament fibroblasts were able to adhere and spread on the 3D wrinkled posts, suggesting that 3D wrinkled surfaces can serve as a physical cue to study cell behavior [16]. In another relevant work, Hu et al. created 3D chromium (Cr) wrinkled surface patterns using a strain mismatch of sputter-coated Cr thin film on pre-strained polydimethylsiloxane micro-ridges, prepared by conventional microfabrication techniques [17]. Systematically varied wrinkle wavelengths were achieved by manipulating the thickness of the thin film deposited on the 3D structure [17]. Although not suitable for use with cells in vitro or in vivo, this 3D wrinkled platform provided a new approach for fabricating curved-shape molds for contact printing, with an increased surface-area-to-volume ratio compared to 2D wrinkling counterparts, and for potential use in wetting and battery applications [17].

The limited work to date on wrinkle formation on 3D surfaces and the accompanying limited understanding of wrinkle formation on 3D surfaces is likely the result, in large part, of the limited fabrication options available. Most of the previously reported 3D wrinkle research used conventional multi-step photolithography that is time-consuming, difficult to automate due to manual processing in which a design-to-prototype cycle takes several days to achieve, and not well-suited to preparing complicated or intricate 3D structures or parts. Moreover, there is a lack of wrinkling research on 3D structures that can mimic the dynamic, in vivo microenvironment, provide physical support and a local environment for mammalian cells, and facilitate tissue development [16,17,18].

To address challenges associated with the study and application of wrinkle formation on three-dimensional (3D) structures—particularly biomimetic and bioinspired cell and tissue scaffolds—the goal here was to develop a simple experimental method to create tunable surface wrinkles on arbitrarily complex 3D structures. To achieve this goal, we developed a low-cost wrinkling approach that combines natural biopolymer silk fibroin (SF) thin films with a recently developed advanced manufacturing technique for trapping (programming) strain in complex 3D shape–memory structures. Using optical and atomic force microscopy, we investigated the effects of programmed strain magnitude, film thickness, and aqueous media on wrinkle stability and morphology, and we used cell culture to evaluate wrinkle cytocompatibility.

## 2. Materials and Methods

### 2.1. Study Overview and Enabling Technologies

To develop a simple experimental method that can create tunable surface wrinkles on arbitrarily complex 3D structures, our strategy was to combine a recently developed 4D printing technique, known as programming via printing (PvP), with a simple dip-coating technique that applies a silk fibroin (SF) biopolymer thin film and thereby enables fabrication of complex structures that, when triggered to undergo shape recovery, form wrinkles upon all surfaces of the structure. The PvP 4D printing technique uses fused filament fabrication (FFF) to 3D-print a shape memory polymer (SMP) [19]. SMPs are a class of smart polymeric materials that can undergo a change from a fixed, temporary shape to a final, permanent shape upon exposure to an external stimulus (e.g., heat, light, pH variation) [20]. In the field of tissue engineering, SMPs as “smart scaffolds” provide unique functionality due to their shape-shifting abilities [21], including self-deployment [22], self-expansion [23], and/or self-fitting [24].

**Figure 1:**
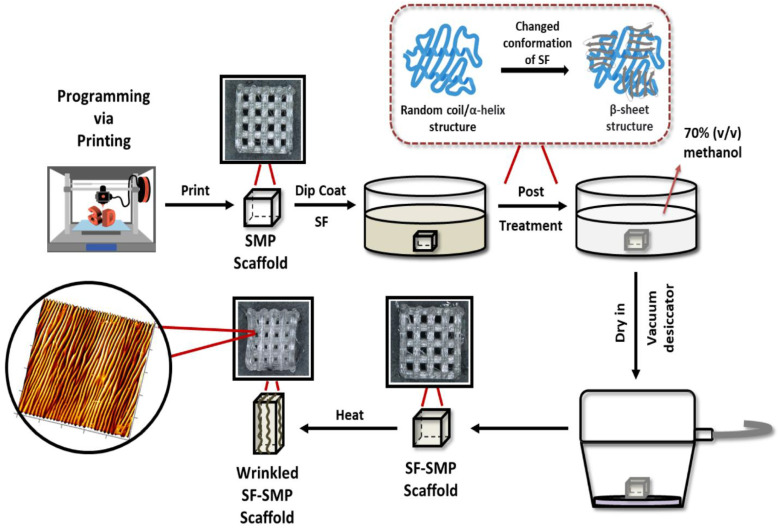
Strategy for creating tunable surface wrinkles on arbitrarily complex 3D structures. The thermo-responsive shape memory polymer (SMP) scaffold is simultaneously fabricated and programmed by programming via printing (PvP). The scaffold is dip-coated with silk fibroin (SF) and then post-treated with methanol to induce a conformational transition from random coil to β-sheet structure in the silk network. To induce shape recovery, the SMP scaffold is heated above its glass transition temperature to recover its permanent shape. Heat-induced contraction of the SMP causes the SF coating to buckle, thereby forming wrinkles on the 3D surface. The representative atomic force microscopy (AFM) micrograph shown is that of an SF-SMP scaffold following recovery that was programmed at 200 °C via printing and coated with a silk concentration of 2% *w*/*v*.

PvP draws (stretches) an SMP filament as it is deposited during FFF to program tensile strains in the printed SMP structure in a fiber-by-fiber fashion, ultimately enabling fabrication and strain programming simultaneously in a single 3D printing process [19]. This approach eliminates the traditional need for a separate programming step following fabrication, and the SMP substrate can immediately undergo shape transformation following printing once exposed to an external stimulus (e.g., heat) [19]. Thus, with PvP, we can enable fabrication of intricate, SMP scaffolds that can undergo heat-inducted contraction to revert to their permanent shape. To acquire thin film wrinkling on intricate 3D structures, an SF thin film was deposited on the surface of PvP-fabricated thermo-responsive shape memory thermoplastic polyurethane (TPU) scaffolds via dip-coating and heat-induced contraction of the SMP used to buckle the SF thin film, thereby producing silk wrinkles throughout the structure (Figure 1). SF is a natural fibrous biopolymer derived from the cocoons of domesticated *Bombyx mori* silkworms that has been widely used as a thin film in the biomedical field. Generally being aqueously soluble, SF can be deposited uniformly on architecturally complex or porous substrates through processes that include spin coating [25], dip coating [26], and molecular self-assembly [27]. SF has been extensively studied for biomaterials’ application, such as tissue engineering, drug delivery, and wound healing, due to its remarkable mechanical properties, tunable biodegradability, biocompatibility, and capacity to support the adhesion, proliferation, and differentiation of various mammalian cell types in vivo [28,29,30,31].

In this study, we sought to develop a low-cost, tunable wrinkling approach by combining SF films with PvP, and we characterized the 3D wrinkle topography using optical and atomic force microscopy to examine the effects of programmed strain magnitude, SF processing variables, and aqueous media on the stability, integrity, and morphology of protein-based wrinkles. As a proof of concept for the development of biomimetic cellular microenvironments that can advance understanding and control of cell behavior in engineering tissue constructs, we studied the cytocompatibility of the wrinkles using murine embryonic fibroblast cells.

### 2.2. Shape–Memory Polymer (SMP) Scaffold Preparation

PvP was used to simultaneously fabricate and program 3D SMP log pile scaffolds so that, when triggered by heat, the scaffold would contract along the direction of the log piles, thereby causing biaxial contraction in the x-y plane (parallel to the build plate) and Poisson expansion in the orthogonal z direction. The longitudinal contraction of the log piles would, therefore, provide controlled compression of the silk thin film and cause formation of generally circumferential wrinkles around the log piles. In our study, we 3D-printed dynamic, shape–memory polymeric (SMP) scaffolds. SMPs are a class of smart polymeric materials that have previously been used to serve as active, compliant substrates to trigger wrinkle formation in vitro [13,14,22,23,24,25,26]. To apply our thin film for bilayer wrinkling, SF is a natural fibrous protein derived from the cocoons of domesticated Bombyx mori silkworms and has been commonly used as a thin film to modify the surface properties of various polymeric substrates to optimize cell behavior, improve biocompatibility, and provide tunable biodegradability suitable for biomedical applications [27,28,29,30,31]. It has also been previously demonstrated to serve as an appropriate biopolymeric thin film to induce wrinkle formation [32]. Given that SF will serve as the thin film, we demonstrate the ability to develop and tune 3D scaffolds that can autonomously actuate silk wrinkles upon heat-induced contraction of the SMP. Commercially available semicrystalline TPU pellets (MM-4520; SMP Technologies, Inc., Tokyo, Japan) with a nominal glass transition temperature (T_g_) of 45 °C were purchased. Based on the manufacturer’s technical sheet, the TPU is comprised of 4,4′-diphenylamine diisocyanate and soft segments of polypropylene glycol. Prior to extrusion into filaments (Composer 450, 3devo., Utrecht, The Netherlands), the TPU pellets were dried for a minimum of 24 h at 50 °C in a vacuum oven to remove any residual moisture. Next, a spool of TPU filament was loaded into a fused deposition modeling 3D printer (Ender 3 Pro., Creality, Shenzhen, China) with a 0.4 mm nozzle diameter for depositing the semi-molten material onto the build plate in a layer-by-layer process. To ensure proper sample adhesion to the build plate, the plate was covered with Kapton tape and maintained at 25 °C. To print the filament into programmed SMP scaffolds (dimensions: 7.5 mm × 7.0 mm × 4.6 mm), 3D log-pile constructs were created in computer-aided design software (Autodesk Fusion 360, San Francisco, CA, USA), and STL files were converted to g-code using slicing software (Ultimaker Cura v. 5.0., Utrecht, The Netherlands). The scaffolds were printed with the logs, alternating at 0° and 90° layer-by-layer for shape memory biaxial contraction. Samples were printed at three nozzle temperatures (200, 210, and 220 °C) with a print speed of 30 mm/s and print flow rate (extrusion multiplier) of 125%. Once printed, samples were stored in a desiccator until use.

### 2.3. Silk Fibroin (SF) Preparation

For the present work, we adapted an approach to biopolymer wrinkling on active, smart materials we recently reported [32]. In the prior study, we employed traditional post-fabrication SMP programming to produce uniaxial micro-and nanosized silk fibroin (SF) wrinkles on 2D thermo-responsive shape memory polymer (SMP) substrates. By manipulating specific system parameters (e.g., film thickness, strain magnitude, shape recovery, temperature), we determined how to control the wrinkle wavelengths and amplitudes on the micron and sub-micron length scale. In the present work, each SMP scaffold was coated with a SF thin film, with the intent being for the SF thin film to buckle into a wrinkled state during SMP shape recovery. Prior to use, a commercially available stock solution of SF (~100 kDa, extracted from the *Bombyx mori* silkworm, Ca# 5154, Advanced Biomatrix, Carlsbad, CA, USA) was centrifuged at 3700 rcf for 30 min to remove any protein aggregates. The 5% *w*/*v* stock solution was stored in a −80 °C freezer prior to use. The final concentration of SF solution was then adjusted to varying concentrations (1, 2, and 3% *w*/*v*) by adding ultrapure water (Millipore Milli-Q water systems, Billerica, MA, USA). To coat the SMP scaffolds with SF, each scaffold was dip-coated in a vial of aqueous silk solution at room temperature, ensuring that the whole surface of the 3D construct was exposed to the suspension. The dipping step lasted for 2 min, after which each scaffold was dipped in a solution of 70% methanol for 5 s to promote silk fibroin coating crystallization (e.g., enhancement of beta-sheet content). Post-methanol treatment samples were dried in a vacuum desiccator overnight.

### 2.4. Characterization of Strain Trapping in Programmed via Printed (PvP) 3D Scaffolds

Strain programming in PvP is controlled via various printing parameters, with nozzle temperature serving as a key parameter [19]. To characterize the influence of nozzle temperature on strain recovery and, therefore, wrinkle formation of scaffolds fabricated via PvP, 3D scaffolds (n = 6 per nozzle temperature) were printed at three nozzle temperatures (200, 210, and 220 °C). The lower limit of the nozzle temperature range (200 °C) was selected to avoid poor fiber bonding, while the upper limit of the nozzle temperature range (230 °C) was selected following preliminary studies to avoid material degradation and bubble formation in the extruded fiber. Following fabrication, the SMP scaffolds were imaged before and after shape recovery with a KH-8700 optical digital microscope (Hirox, Tokyo, Japan) at different magnifications (20×, 40×, 350×, 700×, 1750×, and 3500×).

### 2.5. Characterization of 3D Surface Wrinkles under Dry Conditions

To study wrinkling of SF films under dry conditions, the programmed SF-SMP scaffolds were placed in an isothermal oven at 90 °C to trigger the return of the SMP to its original permanent shape. This specific recovery temperature was selected because, in preliminary experiments, it was identified as a temperature above the dry T_g_ at which complete shape recovery occurred. Following triggering, the presence of the silk wrinkle morphology was confirmed by imaging with a KH-8700 optical digital microscope at different magnifications (20×, 40×, 350×, 700×, 1750×, and 3500×) and scanning electron microscopy (SEM, Jeol JSM 5600, Tokyo, Japan). To quantify the silk wrinkle morphology, a Nano R-2 CA atomic force microscope (AFM; Pacific Nanotechnology, Santa Clara, CA, USA) was used in contact mode with a Si_3_N_4_ cantilever (spring constant: 5 N/m). A 50 μm by 50 μm square area was scanned with a scan rate of 1.5 Hz. The wrinkle wavelength and amplitude were determined using Gwyddion v. 2.60 (Czechia, Europe) and Nanoscope Analysis v. 1.5 (Bruker, MA, USA) software. Silk film thickness was determined using a scratch-and-scan method, in which part of the SF film was removed by scratching it with a razor blade. The scratched area was scanned in AFM contact mode, and the scan rate was fixed to 1.5 Hz on a 50 μm by 50 μm square area. The film thickness was determined by calculating the step-height between the film and bare substrate.

### 2.6. Characterization of Silk Wrinkle Stability in Media

To study the stability of wrinkled SF films in simulated cell-culture conditions (aqueous media at body temperature), varying silk concentrations (1–3%) were dip-coated onto scaffolds printed at 200 °C. The selection of 200 °C was informed by preliminary experiments, which indicated that this nozzle temperature enables high strain recovery to achieve pronounced wrinkle formation on the constructs. Scaffolds were then heated in an isothermal oven at 90 °C to induce silk wrinkle formation. Then, samples were immersed for 24 h in 1 mL of complete medium: Basal Medium Eagle (BME) supplemented with 10% fetal bovine serum (FBS), 1% penicillin/streptomycin (PS), and 1% L-glutamine (L-glut). Optical microscopy was used to image the wrinkled surfaces before and after immersion in media, and AFM was used to characterize the amplitude and wavelength prior to and following media incubation, as previously described for dry wrinkle characterization.

### 2.7. Cytocompatibility of 3D Silk-Wrinkled Surfaces

Cytocompatibility of the engineered silk wrinkled structures was evaluated using C3H/10T1/2 mouse embryonic fibroblasts (ATCC, Manassas, VA, USA), a cell line we have frequently used in the development and application of cytocompatible SMPs [19,33,34,35]. Prior to conducting the experiment, the following samples were prepared: unstrained SMP scaffolds not coated with silk; unstrained SF-SMP scaffolds; and strained SF-SMP scaffolds dip-coated in SF solution and post-treated in methanol (as previously described for the silk coating process). All sample groups were sterilized using UV light in a biosafety cabinet (BSC) for 48 h prior to cell seeding. Cells were cultured in a petri dish with complete medium at 37 °C/5% CO_2_. Once 70% confluency was achieved, cells were dissociated with 0.25% trypsin for 5 min, centrifuged at 160 rcf for 5 min, resuspended with complete medium, the solution was seeded onto each sample in a 24-well plate with a density of 500,000 cells per well and 600 µL of complete medium and incubated at 37 °C/5% CO_2_ for 24 h. To evaluate sample cytocompatibility, live (Calcein AM, green) and dead (Ethidium Homodimer-1, red) stains (Invitrogen, Chicago, IL, USA) were applied to the cell-seeded samples for 30 min at 37 °C prior to fluorescent imaging under an Axiovert inverted fluorescence microscope (Zeiss Microscopy, München, Germany). Three fields of view were imaged per sample, and cytocompatibility testing was performed on three independent samples. Cell viability was calculated by dividing the number of live cells by the total number of cells.

### 2.8. Statistical Analysis

Results are reported as mean ± standard deviation. All experiments were repeated three times (n = 3). One-way ANOVA followed by Holm–Sidak multiple comparisons tests between groups was performed for comparisons involving more than two groups. Student’s *t*-test was used for two-group comparisons. The significance of results is reported as * *p* < 0.05, ** *p* < 0.01, *** *p* < 0.001, **** *p* < 0.0001.

## 3. Results

### 3.1. Strain Trapping within Programmed via Printed (PvP) 3D Scaffolds

**Figure 2:**
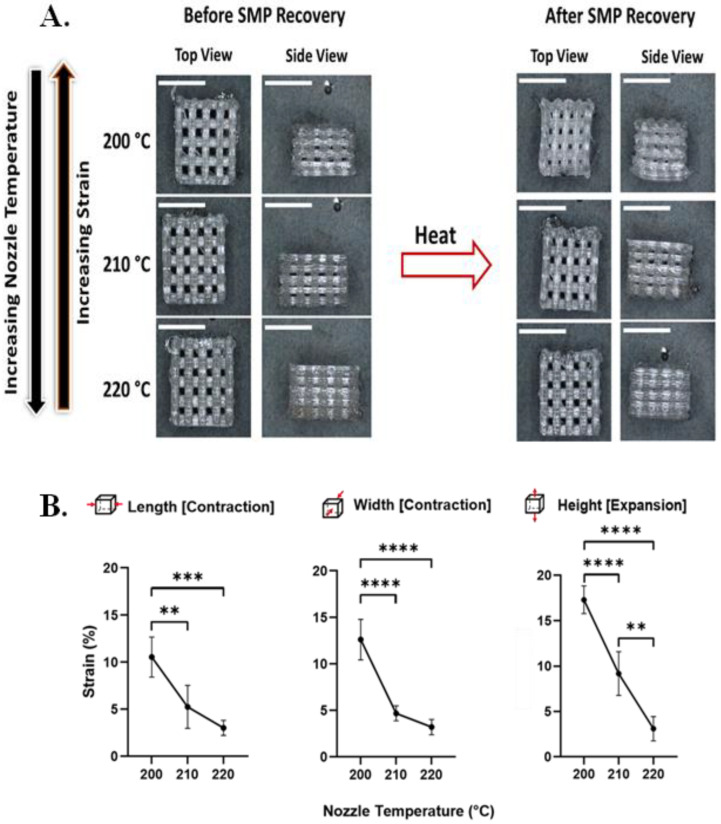
Nozzle temperature affects strain recovery in 3D SMP log-pile scaffolds. (**A**) Representative optical microscopy images of 3D scaffolds before and after recovery when printed at different nozzle temperatures (200 °C, 210 °C, and 220 °C; scale bars = 5 mm). (**B**) Detailed characterization of strain recovered upon triggering as a function of nozzle temperature during printing demonstrates that as the nozzle temperature increases, the programmed strain decreases. (n = 6, one-way ANOVA, followed by Holm–Sidak multiple comparisons test between groups, ** *p*< 0.01, *** *p* < 0.001, **** *p* < 0.0001).

Upon SMP recovery, there was, as intended, a macroscopic change in the bulk shape of the PvP 3D scaffolds (Figure 2A). The length and the width of the scaffolds underwent significant contraction, while there was significant expansion in the height of the substrates due to the Poisson effect. In terms of the effect of print temperature, an increase in nozzle temperature resulted in a decrease in the magnitude of shape change observed upon triggering (Figure 2B). This finding is consistent with previous studies demonstrating the effect of PvP in 3D architectures [19].

### 3.2. Dry Wrinkle Characterization

**Figure 3:**
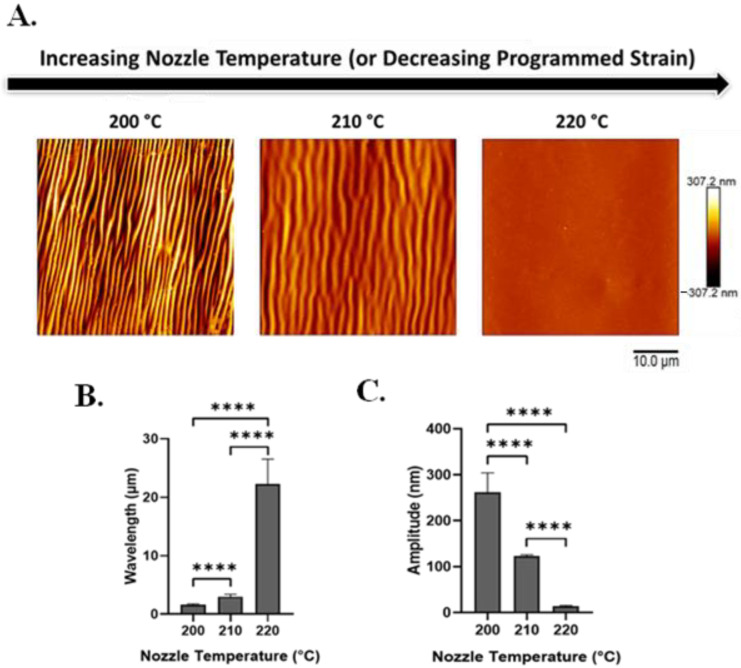
With increasing nozzle temperature (which produces decreased programmed strain), silk wrinkle wavelength increases while wrinkle amplitude decreases. (**A**) Atomic force microscopy images of the 2% silk wrinkled topographies produced, following heat-induced contraction of SMP scaffolds printed at different nozzle temperatures (scale bar = 10 μm). Wrinkle wavelength (**B**) increases and wrinkle amplitude (**C**) decreases as nozzle temperature increases. (n = 3, one-way ANOVA, followed by Holm–Sidak’s multiple comparisons test between groups, **** *p* < 0.0001).

Using AFM and SEM, we characterized the effect of nozzle temperature on wrinkle wavelengths and amplitudes (Figure 3A and Figure S1).

With heat-induced SMP shape recovery, wrinkle wavelength increases as printing temperature increases (and recovered strain decreases). Wavelength increased from 1.6 ± 0.18 to 22.3 ± 4.3 μm with an increase in nozzle temperature from 200 to 220 °C (Figure 3B). In contrast, wrinkle amplitude decreases as the nozzle temperature increases (and recovered strain decreases). Amplitude decreased from 262.2 ± 42.04 to 13.5 ± 2.1 nm with an increase in printing temperature from 200 to 220 °C (Figure 3C).

**Figure 4:**
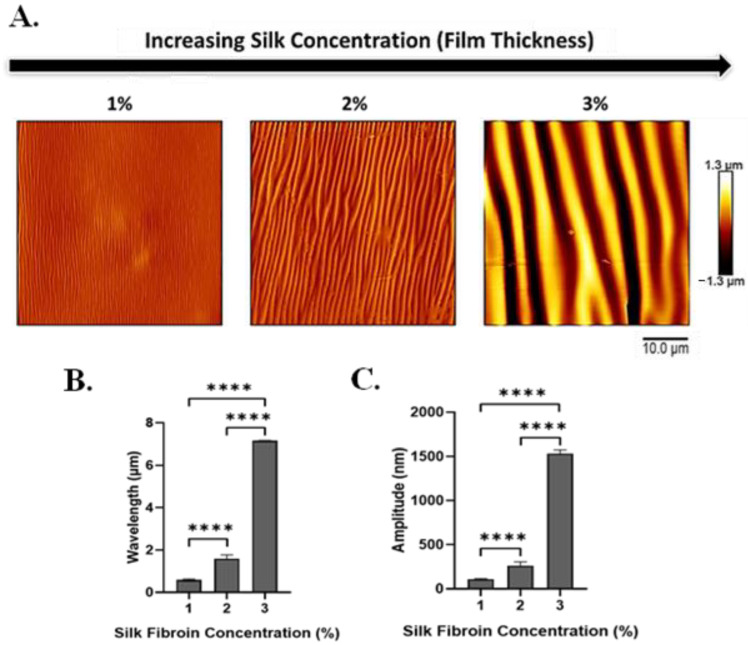
With increasing silk fibroin concentration (which produces increased film thickness), silk wrinkle wavelength and amplitude increase. (**A**) Atomic force microscopy images of the silk wrinkled topographies recovered following heat-induced contraction of the SMP scaffold with different silk concentrations (scale bar = 10 μm). Wrinkle wavelength (**B**) and amplitude (**C**) increase as silk fibroin concentration increases. (n = 3, one-way ANOVA, followed by Holm–Sidak’s multiple comparisons test between groups, **** *p* < 0.0001).

To investigate the effect of film thickness on the silk-wrinkled surface patterns, the SF concentration was adjusted from 1 to 3% *w*/*v* (Figure S2). Silk concentration was found to affect both wrinkle wavelength and wrinkle amplitude (Figure 4A and Figure S3), and the concentration effects were further affected by the nozzle temperature at which samples were printed. Specifically, the wrinkle wavelength and amplitude increase as the SF concentration (and increasing film thickness) increases. Wavelength increased from 0.6 ± 0.04 to 7.2 ± 0.01 μm with increasing silk concentration (Figure 4B), and amplitude increased from 109.4 ± 7.2 to 1534.6 ± 39.4 nm (Figure 4C).

**Figure 5:**
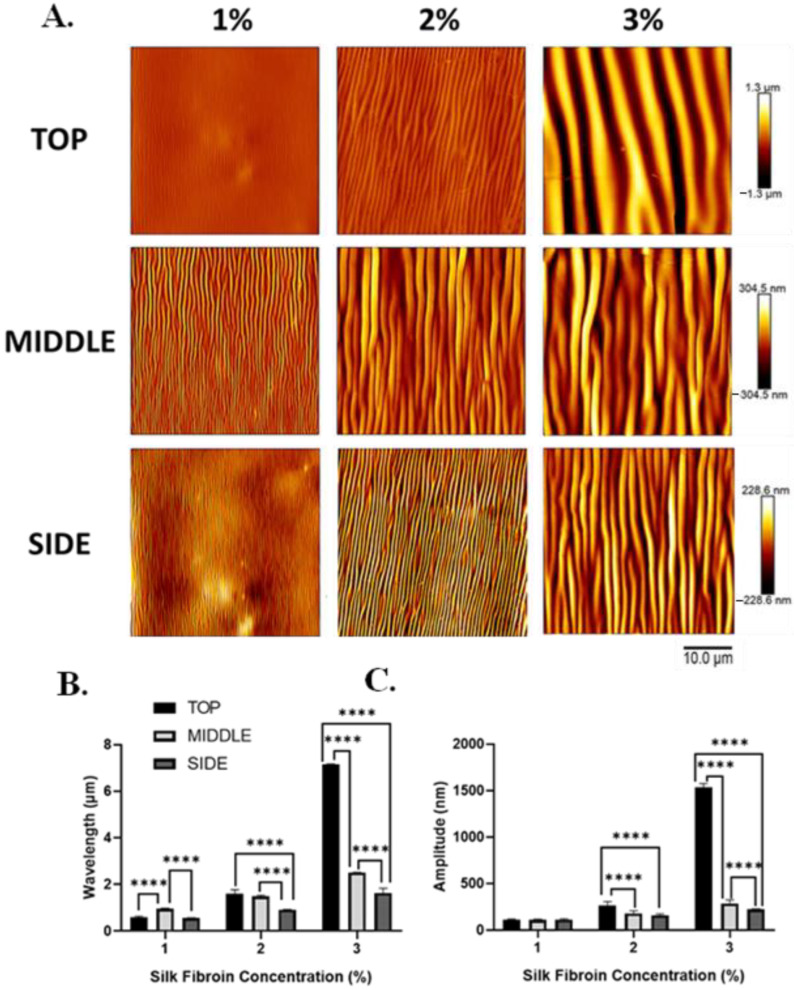
Silk wrinkle wavelength and amplitude increase as the silk fibroin concentration (and, therefore, film thickness) increase in different positions of the scaffold architecture. (**A**) Atomic force microscopy images of the silk wrinkled topographies recovered following heat-induced contraction of the SMP scaffold with different silk concentrations (scale bar = 10 μm). Note that each row has a false-color range scaled to the data present in that row. For presentation of the same data on a single false-color range, please see Figure 6. Wrinkle wavelength (**B**) and amplitude (**C**) increase with increasing silk concentration at the top, middle, and side of the scaffold. (n = 3, one-way ANOVA, followed by Holm–Sidak’s multiple comparisons test between groups, **** *p* < 0.0001).

**Figure 6:**
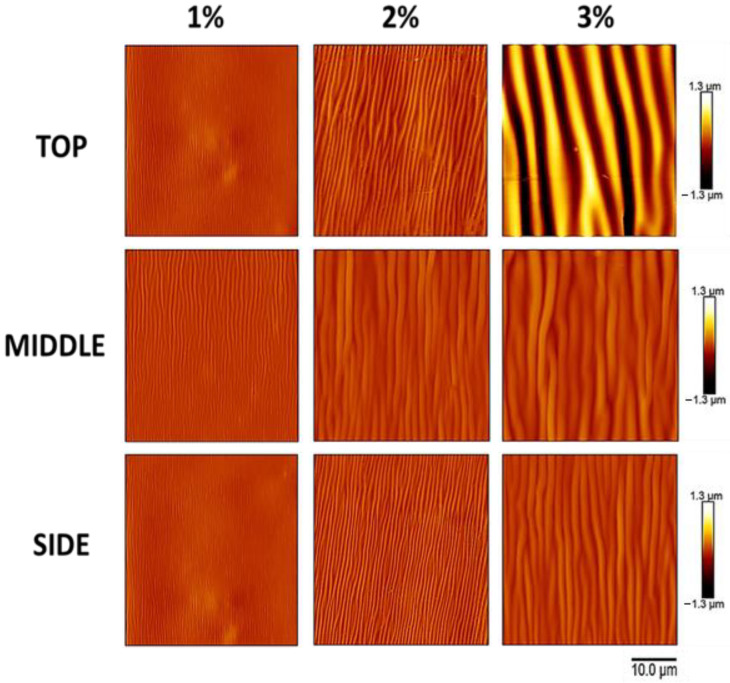
The atomic force microscopy data of Figure 5, presented on a single false-color range, clearly demonstrate the increase in wrinkle amplitude with increasing silk fibroin concentration (which produces increased film thickness) and the differences in wrinkle amplitude at the top, middle, and side of the scaffold. Atomic force microscopy images of the silk wrinkled topographies recovered following heat-induced contraction of the SMP scaffold with different silk concentrations (scale bar = 10 μm). Note that all images have a single false-color range. For presentation of the same data with false-color ranges scaled to the data present in each row, please see Figure 5.

To gain insight into the extent to which wrinkle characteristics vary at different positions in the 3D scaffold architecture, AFM was performed on the top, middle, and side region of the construct (Figure 5 and Figure 6). At all positions within the scaffold (Figure 5A and Figure 6), wrinkle wavelength (Figure 5B) and amplitude (Figure 5C) were found to be affected by SF concentration.

In addition, wrinkle wavelength and, for some silk concentrations, amplitude were found to be affected by the position within the scaffold. For all silk concentrations, wrinkle wavelength was found to vary across positions within the scaffold (Figure 5B). For the 1% *w*/*v* dip-coated scaffolds, the wavelengths at the top, middle, and side positions were 0.6 ± 0.04, 1.0 ± 0.02, and 0.5 ± 0.01 μm, respectively. The wavelengths at the top, middle, and side positions of the 2% *w*/*v* dip-coated scaffolds were 1.6 ± 0.2, 1.5 ± 0.06, and 0.9 ± 0.008 μm, respectively. The wavelengths at the top, middle, and side positions of the 3% *w*/*v* dip-coated scaffolds were 7.2 ± 0.01, 2.5 ± 0.005, and 1.6 ± 0.2 μm, respectively. For 2% and 3% silk concentrations, wrinkle amplitude was found to vary across positions within the scaffold (Figure 5C). The amplitudes at the top, middle, and side regions of the 1% *w*/*v* dip-coated scaffolds were 109.4 ± 7.2, 105.0 ± 9.9, and 111.6 ± 7.1 nm, respectively. For the 2% *w*/*v* dip-coated scaffolds, the amplitudes at the top, middle, and side positions of the 2% *w*/*v* dip-coated scaffolds were 262.3 ± 42.04, 176.6 ± 29.1, and 157.3 ± 14.04 nm, respectively. Lastly, the amplitudes at the top, middle, and side regions of the 3% *w*/*v* dip-coated scaffolds were 1534.6 ± 39.4, 284.7 ± 38.3, and 220.2 ± 5.5 nm, respectively.

### 3.3. 3D Silk-Wrinkled Surfaces’ Stability in Media

**Figure 7:**
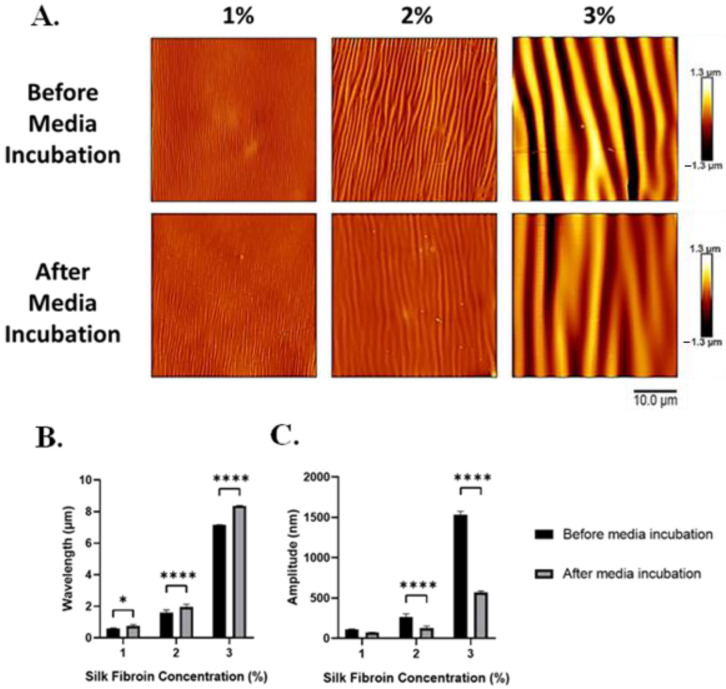
Following incubation in the medium, silk wrinkle amplitude decreased, while wrinkle wavelength increased. (**A**) Atomic force microscopy images of the silk wrinkled topographies prepared via different silk dip-coating concentrations before and after immersion in media (scale bar is 10 μm). Wrinkle wavelength (**B**) increases, while amplitude (**C**) decreases for the wrinkle topographies following immersion in media for 24 h. (n = 3, Student’s *t*-test for two group comparisons, * *p* < 0.05, **** *p* < 0.0001).

To evaluate the stability and pattern of wrinkle morphology in an aqueous environment, the 3D wrinkled scaffolds were submerged in complete medium at 37 °C for 24 h and analyzed by AFM (Figure 7A).

Before media incubation, the wavelengths of the 1, 2, and 3% silk-wrinkled surfaces were 0.6 ± 0.04, 1.6 ± 0.2, and 7.2 ± 0.01 μm, respectively. Following media incubation, the wavelength of the silk wrinkles increased modestly but statistically significantly to 0.8 ± 0.09, 2.0 ± 0.3, and 8.4 ± 0.02 μm (Figure 7B). We attribute the increase in wavelength to the wrinkles spreading as a result of hydration of the SF film. Within a hydrated environment, water molecules can rapidly penetrate the amorphous regions of the silk matrix and act as a plasticizer, which can cause the silk film to undergo a reduction in stiffness (or modulus) and increased plastic deformation [36]. There was also a large reduction in the amplitudes of the silk-wrinkled surfaces. Prior to media incubation, the amplitudes of the 1, 2, and 3% silk wrinkles were 109.4 ± 7.2, 262.3 ± 42.04, and 1534.6 ± 39.4 nm, respectively. Following hydration, the amplitudes of the silk wrinkles decreased to 70.8 ± 5.6, 128.2 ± 23.8, and 568.5 ± 18.9 nm (Figure 7C).

### 3.4. Cytocompatibility of 3D Silk-Wrinkled Scaffolds

**Figure 8:**
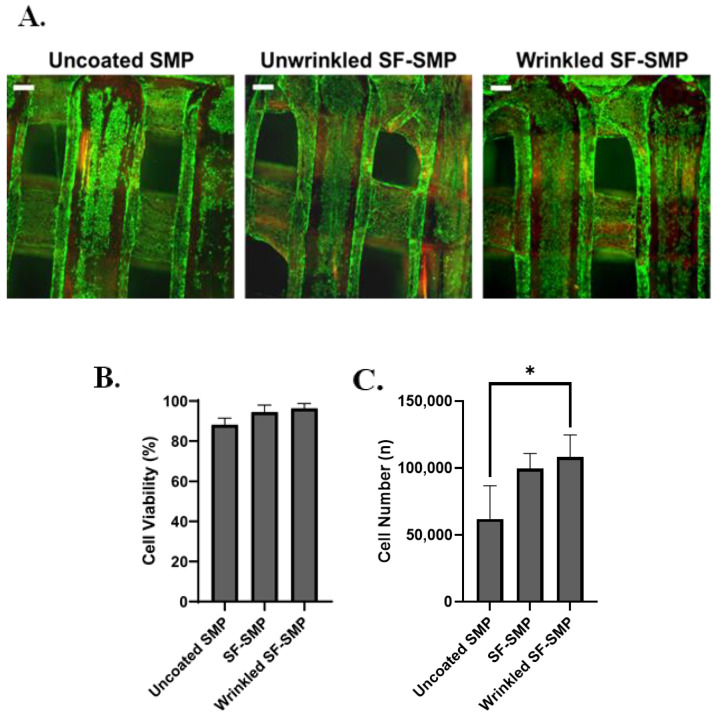
Silk wrinkles displayed high cell viability and adhesion. (**A**) Representative fluorescent images of live (green) and dead (red) C3H/10T1/2 cells after 24 h for uncoated shape memory polymer (SMP) scaffold, unwrinkled SF-SMP scaffold, and wrinkled SF-SMP scaffold (scale bar = 100 μm). (**B**) Quantitative results of cell viability and (**C**) cell number of calculated attach cells (n = three samples, with three area images per sample, one-way ANOVA, followed by Holm–Sidak multiple comparisons test between groups, * *p* < 0.05).

When cultured on 2% silk-wrinkled scaffolds, C3H/10T1/2 cells displayed high cytocompatibility comparable to that observed on an unwrinkled SF-SMP scaffold and an uncoated SMP control scaffold (Figure 8A). The engineered silk-wrinkled scaffolds displayed 96.3% cell viability as compared to the uncoated (88.2%) and unwrinkled (94.5%) controls (Figure 8B), suggesting the 3D silk-wrinkled scaffolds’ suitability for use in cell-based applications. Additionally, more cells were able to adhere and spread on the wrinkled scaffold compared to the uncoated controls (Figure 8C). Although there was no significant difference found between the wrinkled and unwrinkled SF-SMP scaffolds, there was a significant difference between the silk-wrinkled scaffold and uncoated scaffold.

## 4. Discussion

Here, we have demonstrated a strategy for functionalizing and tuning the surface topography of complex 3D substrates using functional protein wrinkled coatings. This was achieved by combining natural SF thin films with a recently developed PvP advanced manufacturing technique for trapping (programming) strain in complex 3D shape–memory structures. In contrast to conventional micro/nanofabrication methods, additive manufacturing offers a simple, attractive alternative. Not only can 3D printers transform designs into functional prototypes in hours, but users have the ability to customize and fabricate intricate and complex geometries that would be challenging or impossible using conventional soft lithography approaches. 3D printing also enables the consistent production of parts with similar dimensions and surface finish when used [37,38]. Moreover, the most widely studied or used biomedical applications of additive manufacturing technologies include the fabrication of scaffolds for tissue engineering and regenerative medicine [39,40,41], the development of customizable prosthetics and implants [42], and wound dressings [43].

In studying this application of PvP, we varied the nozzle temperature during 3D printing, thereby controlling the magnitude of programmed strain of the SMP, and we found that programmed strain has a significant effect on the silk wrinkle wavelength and amplitude. As observed in prior work [19], we found that as nozzle temperature increases, the strain recovery (or contraction) of the SMP decreases, enabling higher wavelengths and lower amplitudes to be achieved. We found that lower nozzle temperatures (200 °C) programmed a higher strain magnitude into the SMP, enabling the scaffold to undergo pronounced shape contraction to induce silk wrinkle formation on the surface of the scaffolds. In contrast, higher nozzle temperatures (220 °C) programmed a lower strain magnitude into the SMP, resulting in insufficient shape contraction and the absence of silk wrinkling on the surface of the scaffolds. Van Manen et al. [44] reported that the effect of nozzle temperature on strain deformation of printed constructs can be attributed to the combined influence of the molecular orientation along the direction of the printing path and the cooling history of the polymer. They found that lower printing temperatures were found to increase filament shrinkage during printing. This is due to the drawing of the fibers deposited from the nozzle, as the nozzle moves and the SMP cools, enabling it to undergo stretching [44]. Thus, lower printing temperatures create a more viscous fiber, which, when stretched, can store more strain energy than the less viscous fiber produced at higher printing temperatures.

Collectively, our dry wrinkle characterization results demonstrate an increase in silk wrinkle wavelength and amplitude with increasing silk concentration, which is due to the increase in film thickness. When conducting the dip-coating technique, the thickness of the deposited film can be modulated by the number of dipping cycles and the suspension concentration. This study focused on modulating the thickness of the SF film by increasing the silk concentration during dip coating, which leads to an increase in film thickness. We observed varying film thickness by manipulating the silk concentrations from 1 to 3% *w*/*v* (Figure S2). From the bilayer buckling theory reported by Chang et al. [45], the relationship for wavelength, amplitude, and critical strain required to create wrinkles for small deformation is given as follows:

(1) $$\lambda =2\pi h_{f}{\left(\frac{{\bar{E}}_{f}}{3{\bar{E}}_{s}}\right)}^{\frac{1}{3}}$$

(2) $$A=h_{f}{\left(\frac{\varepsilon }{\varepsilon_{c}}-1\right)}^{\frac{1}{2}}$$

(3) $$\varepsilon_{c}=\frac{1}{4}{\left(\frac{3{\bar{E}}_{s}}{{\bar{E}}_{f}}\right)}^{\frac{2}{3}}$$

where *λ* is the wavelength, *h_f_* is the film thickness, *Ē_f_* is the plane-strain modulus of the film, *Ēs* is the plane-strain modulus of the substrate, *A* is the amplitude, *ε* is the applied compressive strain, and *ε_c_* is the critical strain (minimum strain) needed to induce buckling. The relationship between the silk concentration and silk wrinkle characteristics observed in our results indicate that as the thickness of the SF film increases due to increasing silk concentration, the wrinkle wavelength increases, in agreement with Equation (1). Additionally, in agreement with Equation (2), the wrinkle amplitude also increases with the increasing film thickness due to the increasing silk concentration. Additionally, we found that the silk wrinkle amplitude increases with decreasing nozzle temperature (or increasing strain deformation), in agreement with Equation (2). We further observed that the wavelength decreased with decreasing nozzle temperature. This result complements our previous study [11], in which a 2D gold-SMP wrinkling bilayer system demonstrated a decrease in wrinkle wavelength with increasing programmed strain.

When we examined the extent to which wrinkle characteristics vary at different positions in the 3D scaffold architecture, we found that, for all silk concentrations, silk wrinkles on the sides of the scaffold had lower wavelengths (roughly below 2 μm) compared to those at the top (roughly below 7 μm) and middle (roughly below 3 μm) positions of the scaffold. In addition, at the top position of the scaffolds, we observed larger silk wrinkle amplitudes for all silk concentrations, as compared to the middle and side positions of the scaffold. We believe these results can be attributed to constraints that could influence the wrinkling behavior at different positions in the scaffold architecture. The first is a geometrical constraint with respect to the structure of the log-pile scaffold. Since the scaffold contains many welding (or log-to-log bonding) points, the connection of each log to its orthogonal neighbor can be anticipated to act as an anchor that locally constrains shape–memory strain recovery at the surface of the logs near the weld. Although average strain recovery along all printed logs appears to be fairly consistent throughout the scaffold, as demonstrated by the uniform macroscopic biaxial contraction of the scaffolds, the presence within the scaffold of weld points can nevertheless be anticipated to create local areas of non-uniform deformation and, therefore, local differences in wrinkle properties. These local effects would most likely be observed at the middle and side positions we studied, where weld points exist above and below the regions analyzed. In contrast, the logs studied at the top position of the scaffold did not reside near any weld points, and thus we expect those logs to be less likely to demonstrate effects of localized constraint, which is consistent with the wrinkles at the top position being most distinct from those at the side and middle positions. Second, we also expect that the dip-coating process may contribute to the observed heterogenous wrinkles of varied wavelengths, amplitudes, and morphologies at all positions of the scaffold for each silk concentration. The observed variations may be due to the challenge of uniformly depositing silk in the interior of an intricate and architecturally complex porous structure. In addition, due to the relatively large volume of solvent used during dip-coating, thicker films would require longer drying times, which may lead to uneven film distribution that could affect the wrinkle characteristics and morphology on the scaffolds [46].

Collectively, these observations suggest both challenges and opportunities in applying the strategy we have reported. For applications in which uniform wrinkle wavelength and amplitude throughout a complex structure are desired, optimization of structure architecture, PvP strain programming, and silk coating may be required. However, for applications in which it would be desirable to tailor wrinkle properties in different regions of a structure, the findings suggest the feasibility of such tailoring through manipulation of architecture, strain programming, or silk thin-film properties.

Previous studies have reported that functionalizing the surface of 3D scaffolds plays a pivotal role in optimizing and controlling the cellular response of mammalian cells cultured in artificial 3D microenvironments [4,47]. Although we focused on viability and adhesion, many 2D wrinkling platforms have been demonstrated as useful tools for dynamically controlling cell alignment [8,9,10,11,12,33,34,48]. In addition, although the present work focused on in vitro study, prior in vivo studies of wrinkled surface topographies suggest the in vivo potential of the strategy studied herein. Wang et al. [49] surgically implanted wrinkled and flat materials in the subcutaneous space of C57BL/6J mice. They found that wrinkled materials prompted macrophages to exhibit higher arginase-1 expression and also promoted blood vessel formation. This indicates that surface wrinkles contribute to alternative activation of macrophages and can potentially be used to modulate the foreign body reaction to implants [49]. Furthermore, Izawa et al. [50] investigated the biocompatibility of wrinkled chitosan (CS) films by implant placement in C57BL/6 mice. Following in vivo evaluation, many neutrophils, fibroblasts, and collagens were observed on the wrinkled CS films. Although many fibroblasts and collagen were observed in the flat CS films, fewer neutrophils were present. The results of the study found that wrinkled CS films induced neutrophil infiltration and the migration of fibroblasts compared to flat CS films, indicating that wrinkled CS films are effective in enhancing wound healing processes. Based on previous studies investigating the influence of wrinkled structures on biological response in vivo, we would anticipate similar findings with our 3D silk-wrinkled surfaces due to the high biocompatibility that SF displays and its ability to affect cell behavior.

To conclude, the morphologies of the 3D silk-wrinkled surfaces were preserved following cell-culture media incubation for 24 h. Our results demonstrated increased silk-wrinkle wavelengths due to hydration of the SF film, which can be attributed to plasticization of the SF by water. Since silk is a biopolymer, water has been previously reported to influence the amorphous and crystallite domains of the silk network [36]. In particular, water acts as a plasticizer for the amorphous domains of silk, which aids in enhancing chain mobility in the amorphous region and minimizes brittleness of silk fibroin. On the other hand, using molecular dynamics simulation on a β-sheet crystallite (which primarily dictates the strength of silk) of *Bombyx mori* silk, it has been previously reported that water weakens the strength of the β-sheet crystallite domains of silk [36]. Cheng et al. reported that water molecules play a weakening role in the formation of hydrogen bonds between β-chains, ultimately reducing the stability of the β-sheet crystallite [36]. This understanding regarding the effect of water in the silk network provides context on how the 3D silk wrinkles are susceptible to molecular changes that could affect its stability and morphology when hydrated.

## 5. Conclusions

We have reported a tunable on-demand 3D SF-SMP bilayer wrinkling platform. The 3D silk-wrinkle topographies were successfully demonstrated under both dry conditions and cell-culture conditions and characterized by AFM and optical microscopy. We found that increasing SMP programmed strain magnitude (or nozzle temperature) increases wavelength and decreases amplitudes of silk-wrinkled surfaces, while increasing silk film thickness increases wavelength and amplitude. For applications in which it is essential to tune wrinkle characteristics in distinct regions of a complex, biomimetic structure, our findings suggest the feasibility of tuning silk-wrinkle wavelengths and amplitudes by modulating the fabricated architecture, strain programming, or silk-film processing variables. Importantly, all silk wrinkles prepared with varying silk concentrations maintained their wrinkles after 24 h in complete media and displayed high cell viability. This novel bioinspired strategy demonstrates potential for the development of biomimetic cellular microenvironments that can progress knowledge and control of cell–material interactions in the development and clinical translation of engineering tissue constructs.

## Data Availability

The raw data supporting the conclusions of this article will be made available by the authors on request.

## Supplementary Materials

The following supporting information can be downloaded at: https://www.mdpi.com/article/10.3390/polym16050609/s1, Figure S1: Scanning electron microscopy (SEM) images of the scaffold architecture for the uncoated SMP, unwrinkled SF-SMP, and wrinkled SF-SMP. Figure S2: Silk concentration controls the thickness of the deposited silk film. Figure S3: Detailed atomic force microscopy (AFM) characterization of the effects of nozzle temperature and silk concentration on the silk wrinkle morphologies.
