# Supplementary material for: Tuning the Topography of Dynamic 3D Scaffolds through Functional Protein Wrinkled Coatings
Source: Polymers (Basel). 2024 Feb 23;16(5):609. doi: 10.3390/polym16050609 (PMC10934732; doi:10.3390/polym16050609)
Supplement: Supplementary file 1 [file polymers-16-00609-s001.zip › polymers-2848867-supplementary.pdf]

SUPPORTING INFORMATION

Tuning the Topography of Dynamic, 3D Scaffolds through Functional Protein Wrinkled Coatings

Elizabeth Oguntade[a,b], Daniel Fougnier[a,b], Sadie Meyer[a,b], Kerrin O'Grady[a,b], Autumn Kudlack [a,b], James H. Henderson[a,b]

[a]Department of Biomedical & Chemical Engineering, Syracuse University, Syracuse, NY 13244

[b]BioInspired Syracuse: Institute for Material and Living Systems, Syracuse University, Syracuse, NY 13244

Corresponding author:

E-mail: jhhender@syr.edu

TABLE OF CONTENTS

Index	*Page*
**1. Additional Supporting Figures**	S3
**Figure S1 –** Scanning Electron Microscopy (SEM) images of scaffold architecture	S3
**Figure S2 –** Varying SF Film Thickness based on different silk concentrations.	S4
**Figure S3 –** Array of tunable silk wrinkle morphologies achieved via manipulating the nozzle temperature and silk concentration.	S5

2. ADDITIONAL SUPPORTING FIGURES

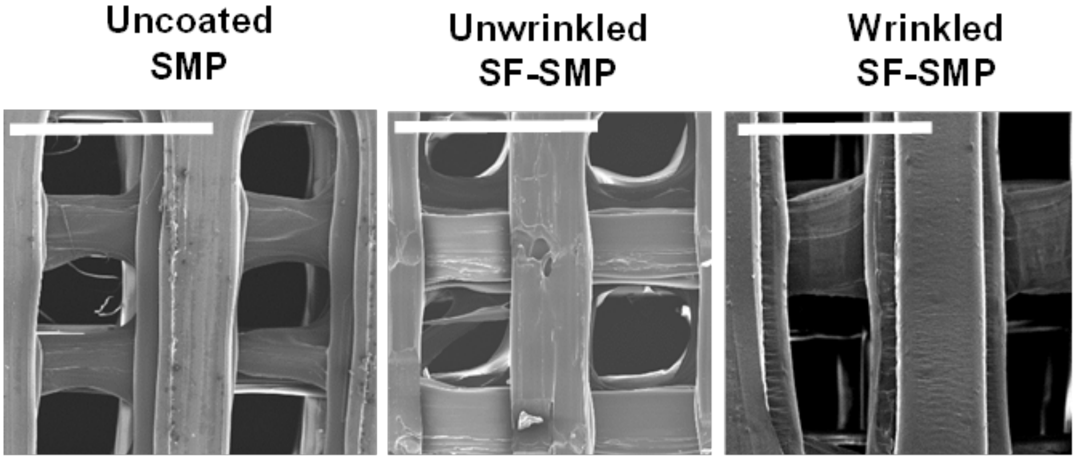

Figure S1. Scanning electron microscopy (SEM) images of the scaffold architecture for the uncoated SMP, unwrinkled SF-SMP, and wrinkled SF-SMP. (Scale bar = 500 μm).

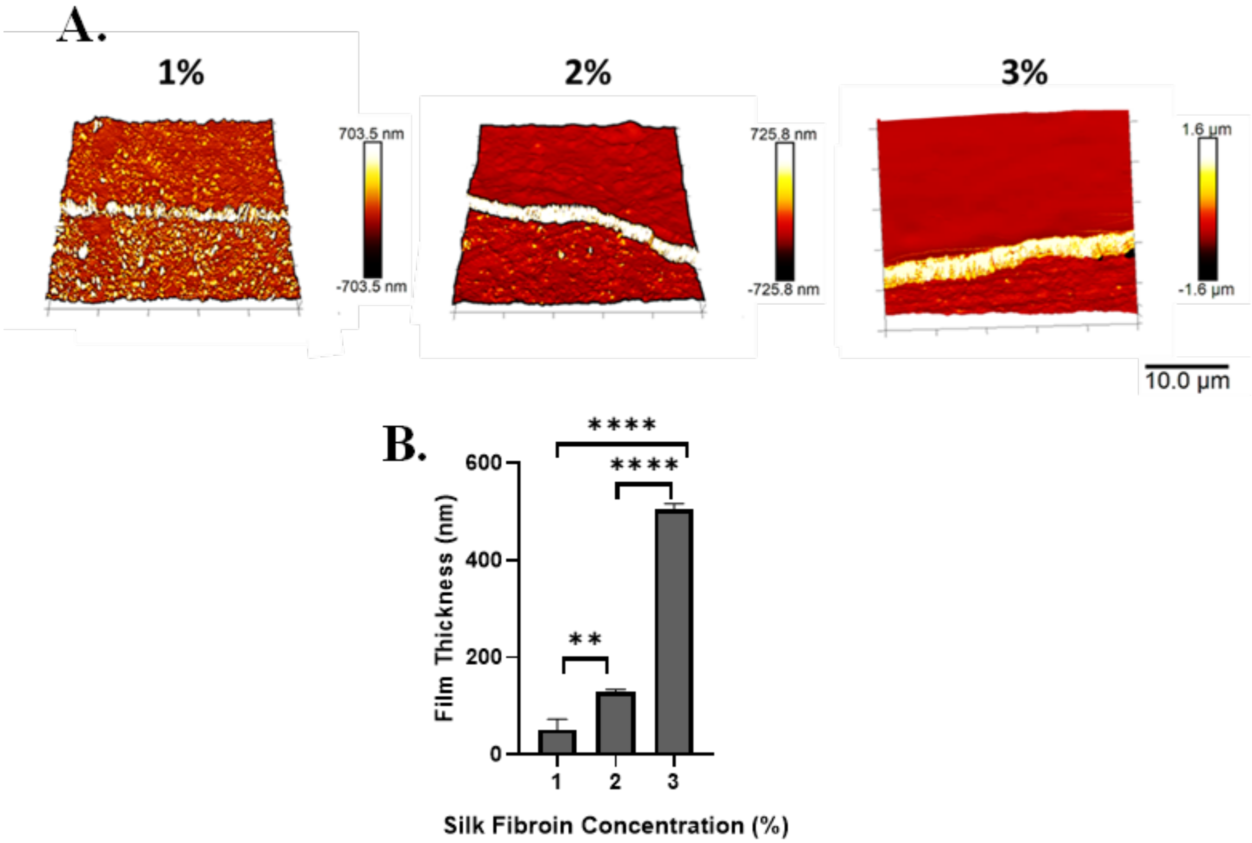

Figure S2. Silk concentration controls the thicknesses of the deposited silk film. (A) Atomic force microscopy images of the silk films with varying dip-coating concentrations. (B) Silk film thickness increases as silk fibroin concentration increases. (n=3, one-way ANOVA, followed by Holm Sidak's multiple comparisons test between groups, *$p< 0.05$, **$p< 0.01$, ***$p<0.001$, ****$p<0.0001$).

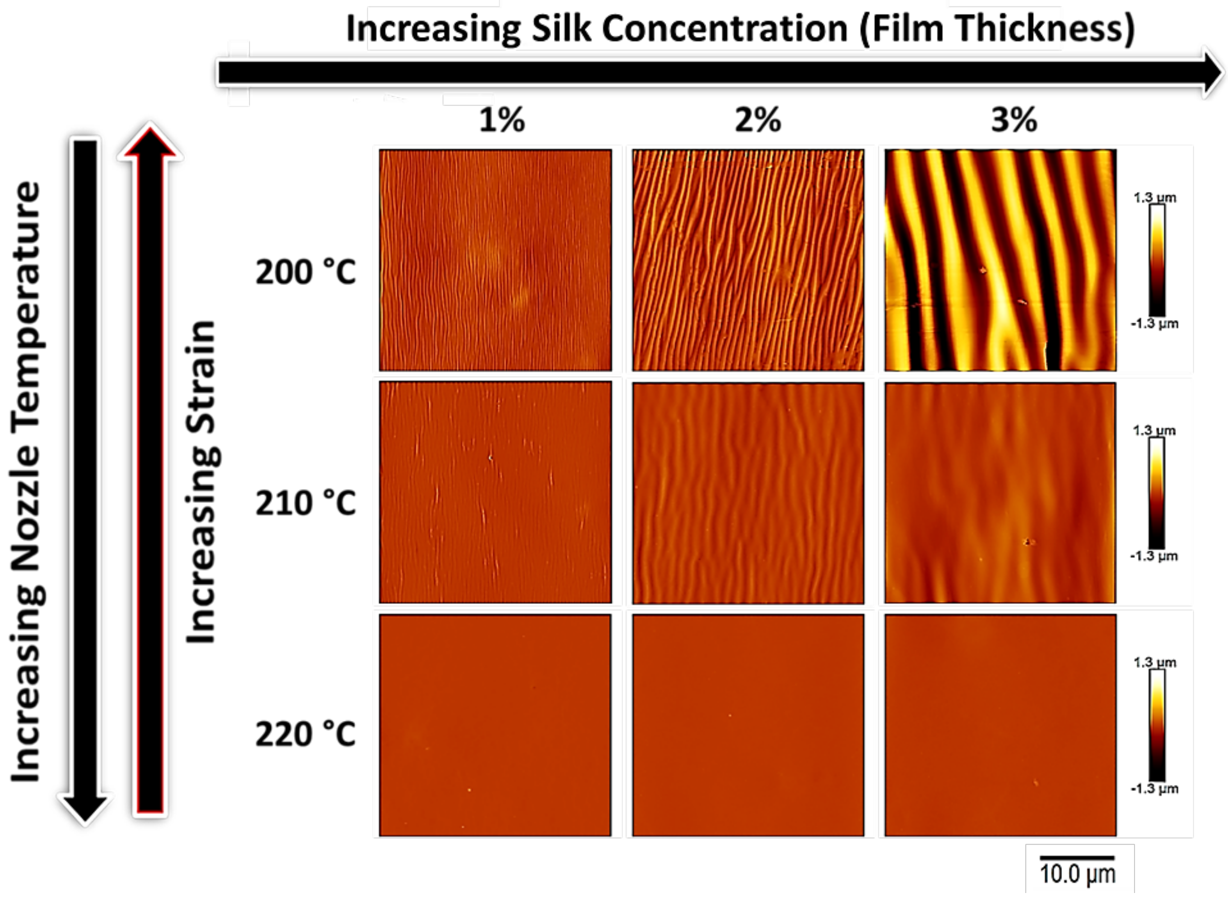

Figure S3. Detailed atomic force microscopy (AFM) characterization of the effects of nozzle temperature and silk concentration on the silk wrinkle morphologies.
